# My Experience with the Dental Pulp

**Published:** 1888-04

**Authors:** Wm. A. Pease

**Affiliations:** Dayton, O.


### My Experience with the Dental Pulp.

BY WM. A. PEASE, DAYTON, O.

(Read before the Mississippi Valley Dental Association.)

More than fifty years ago, when I became a dentist, it was the practice when we wished to insert a pivot tooth, now called a crown, and when the pulp became exposed to destroy it by thrusting a properly prepared hickory splint, or oftener a flexible steel instrument as deeply into it as it would go, turn it rapidly around and therewith excise the pulp. It was also the custom when necessary to destroy the pulp before plugging to open the cavity, especially on the approximal face and then use a hickory splint in the same way, but oftener a piece of East India bamboo, sawed off at each joint, was split off of the required size, properly fitted, often soaked in water a few minutes to make it more tough and flexible, always leaving the outside of the bamboo uncut, as being the least liable to break, and always in an approximate cavity turning that towards the back side before thrusting it down.

It was a barbarous practice tempered with a full equivalent of mercy, as there were no afterclaps; no obstreperous flux to that point, causing tumefaction, a signal that nature had been outraged, to end which she had established a natural drainage tube, and an interrupted series of greater or lesser tumefactions and paroxysms of pain. All of these were then unknown; people enjoyed their pivot teeth; often fastened by a pivot of bamboo, the outside never removed, and that always placed on the lingual face of the root as being the least liable to break. They were fully as enduring as the more complicated crowns of to-day, and then getting a fee that enabled us to adjust the crown to the root, and obliged to make many of our instruments we brought to it much manual and artistic skill. They were nearly, and often, fully as cleanly as the crowns of to-day, resting on an intervening phosphatic layer which are often sprung soon after insertion enough to admit more or less of uncleanliness. Fitted as they were then with an intervening layer of phosphates of to-day, I have no hesitancy in saying they were and would be more enduring than the average crowns as now used, and they were vastly easier to replace in case of an accident. I have positive knowledge that many crowns I inserted lasted more than twenty years; but these were subsequent and are only mentioned to show that careful manipulation under the old methods produced good results. Then everything was secret; there were no dental journals, no colleges, every practitioner guarded his methods, many of them elaborated from his inner consciousness, and valuable, and it was not long before I discovered that others had modes of practice in some respects superior to mine, which were so valuable they would not part with them for love nor money. In vain I tried to fill teeth held in a vise equal to theirs. Their methods I must have, so I dug out a filling, went a long distance, found the dentist, was very inquisitive and ignorant and very nervous, made him explain his method, show his instruments; and at last in the chair, I held his hand mirror and saw his operation. This was several times repeated with others. Once I met, subsequently, one of them at a dental meeting, and when introduced watched to see any sign of recognition, but he had a polite control over tongue and face, or he did not recollect me.

It was some time before I learned the use of arsenious acid. Then the pulps were deadened, but not removed; they, with whatever of arsenic they had absorbed, were sealed in, and so far as I now recollect the percentage of ulcerations was not materially greater than the average complicated operations of to-day; but they were greater than where the pulp was destroyed by mechanical means and immediately removed, in order to set a crown. Well do I remember the first complaint that an incisor tooth had become pink. There it was, immediately over the pulp, as near as the two different structures would admit, a bony ecchymosis; but I soon found if the cavity was opened and the pulp removed the color disappeared. It is now a question with me whether the rather liberal use of arsenic then caused more to be absorbed and to be sealed in with the pulp to ultimately extend through the foramen or ulceration was caused by germs reaching it through the circulation. It is probably the first, as arsenic would not be very palatable to germs, and Fowler's solution might now in careful hands be valuable. It should be known that Fowler's solution by heat can be indefinitely strengthened by taking up more arsenic and still remain clear and will easily penetrate where the paste will not reach, and that a small drop of eight per cent. cocaine applied directly to the pulp will, after five minutes, so numb it that the arsenic will get a painless hold of it. It was some years later that Dr. Solymon Brown published in an illustrated article in the first American Dental Journal his mode of practice. That Journal cost one dollar and a quarter a number. I have it yet; it cost to get modes of practice then, and he was censured for giving the secret away. From the beginning there were teeth suitable for a crown, in which the nerve was dead. After these had been cut down and drilled there was little space left for germs; but it was the custom to run a smaller drill further down to make them less offensive; into which a little cotton dipped in wood kreosote was placed, and when the pivot was thrust in a part was pressed through the foramen. It should be recollected that the kreosote of commerce and carbolic acid are quite modern. I still keep wood kreosote by me; coming down to modern practice I have tried the various remedies or germicidal root dressings, but still think the one I have used for several years the best, the formula of which was: ℞. Potass. Iodide, 1 drachm, Aqua, 1 oz. ft. sol. After an offensive tooth has been prepared and the nerve canal opened as far as practical a broach may be pushed down, the dam being on, or other means employed to keep the cavity from being flooded, when a drop of that solution may be placed in an inferior tooth and almost instantly the patient will start from a slight pain, the fluid having passed the foramen. Here a pretty strong solution of iodide of potassa as limpid and clear as water, passes without trouble to the operator, through the entire canal and is absorbed by capillary attraction into the more or less empty spaces of the fibrils into the cementum and if the buccal roots of the superior molars are opened as far as convenient and a little cotton wet with it is pressed in and an instrument a little larger than the cavity is pressed on it, most of the canals will be filled either by pressure or absorption. In addition to this, if desired carbolic acid or the nasty and offensive smelling iodoform can be used. In regard to iodoform if confined with a cork it is apt to shrink and the iodine to evaporate and to be of very uncertain strength, while a solution of potash and iodine lose little or nothing. Much more might be said in favor of this, how the potash unites with animal matter, and iodine stimulates the absorbents, but these were discussed in the Dental Register several years since.

Thus far the salient points in the treatment of the dental pulp and its remains have been noticed; a few words as to the modern treatment must suffice. This may be divided into two parts, viz.: When the pulp has been suddenly and unexpectedly exposed in operating, and where it has been encroached upon by disease until it has become irritable, and has ached occasionally for a longer or shorter time. The oxychlorides generally killed the pulp; the phosphates were a little better, but both were irritants, and it seemed to me irritants were not indicated for so sensitive an organ as the dental nerve. Both were moderate conductors, and where it survived there would be often periods of pain from thermal change or other cause for months. My first departure was for a young lady æt. 16, whose six year molar was aching and had occasionally ached before—crown cavity, no periosteal complication. The cavity was prepared, point of exposure located; means adopted to prevent flooding, when a drop of wood kreosote was placed on the spot, then a suitable quantity of creta præp was carefully placed over the point of exposure; a cap of tea lead placed on that, and the cavity filled with amalgam, and the pain ceased, and for several years there was no recurrence. My present practice is to touch the point of exposure with a little of an eight per cent. of cocoaine; wait till it begins to take effect, then place on that a little morphine in substance and finish as before. The cocoaine subdues irritation for an indefinite time, and the more persistent morphine quiets it for weeks. Both are strong remedies and must be used with caution, the cocoaine being a non-irritant. It has always seemed unphilosophical to me to use an irritant on so sensitive a tissue as the dental pulp. Creta præp, comes the nearest to my ideal of any substance, a non-conductor, non-irritant; merely homogeneous with the walls of the tooth, it is better in many respects than the same quality of phosphate of lime. It is sometimes a little difficult to adapt to an approximal cavity. My method is this: Prepare the cavity and locate the point to be capped; touch that with a little eight per cent. cocoaine, keep otherwise dry, then cut a piece of tea lead to suit the place, put a piece of gum demar, or bleached shellac; warm a globe ended instrument, press it on the lead and the gum will adhere to it; moisten the place with cocoaine or a little carbolic acid so the chalk will adhere to it and be a little pasty, on to this place a little morphine in substance and apply, then touch a warm instrument to the place over the gum under the lead and it will adhere if the tooth is dry, then press very lightly the edges of the lead to the walls of the tooth; when the tooth can be filled with amalgam or the cap can be covered with phosphate and filled with gold. Sometimes it will be more convenient to apply the paste to the spot with a delicate spatula and then cover with the lead, the space between teeth being small. When a large cavity is to be filled with gold phosphate, or better still amalgam, should be filled over the cap and pressed thoroughly to the walls and base, except over the point of exposure, where it should be lightly touched, or arched. If filled wholly with amalgam then the chalk will not yield under pressure, and the filling settle and leak. I have become so accustomed to this practice that I take great risks.

DISCUSSION.

*Dr. Watt:* Dr. Pease speaks of iodide of potassium. While it remains potassium-iodide it does no more good than so much water; but it is a loose combination and is easily disengaged; the free iodine acts along the tract and kills the "bugs." This is why iodide of potassium is so good a blood purifier. It is a wonderfully perfect chemical mixture on account of its possessing so feeble a combination.

*Dr. Wright:* I would like to ask Dr. Watt if iodide of potassium acts in this way when topically applied?

*Dr. Watt:* Yes; it is disengaged as soon as it comes in contact with animal matter.

